# Molecular characterization of class 1 integrons and gene cassettes in multidrug resistant (MDR) *Klebsiella spp*. isolated from hospitalized and outpatients in Iran, 2009

**Published:** 2013-03

**Authors:** Himen Salimizand, Fereshteh Shahcheraghi, Enayatollah Kalantar, Farzad Badmasti

**Affiliations:** 1 Department of Microbiology, School of Medicine, Kurdistan University of Medical Sciences, Sanandaj, Iran; 2 Department of Microbiology and Microbiology research center, Pasteur Institute, Tehran, Iran

**Keywords:** *Klebsiella spp.*, multidrug resistante (MDR), class 1 integrons

## Abstract

**Background and objectives:**

*Klebsiella* species are of the most common bacteria involved in nosocomial and urinary tract infections. Genetic elements such as class 1 integrons have an important role in the resistance development. In this study, the share of class 1 integrons, the genetic characterization of the integron cassettes and PFGE profiles of the clinical *Klebsiella* isolates are evaluated in Besat University hospital of Sanandaj, Iran.

**Methods:**

Isolates from 17890 clinical specimens were identified by API20E. Antibiotic susceptibility testing and MIC were done for MDR isolates. For investigating class 1 integrons and gene cassettes, PCR by *intI1* integrase and 5’-CS/3’-CS were performed. Integrated gene cassettes were analyzed by PCR-RFLP and sequencing. Pulsed-Field Gel Electrophoresis was carried out for studying of clonality outbreak of isolates.

**Results:**

Thirty five *Klebsiella spp*. were isolated and included 29 *K. pneumoniae* and 6 *K. oxytoca*. All the isolates were susceptible to carbapenems while other antibiotics showed high resistant profile. In all *Klebsiella spp*. PCR for *intI1* integrase and 5’-CS/3’-CS were positive (100%). Sequencing for prevalent bands of internal variable regions between 5’-CS/3’-CS showed *arr-5*, *orfD-aacA4* and *aad5- dfrA17*. PFGE Analysis showed 18 clusters in *K. pneumoniae* with clonality relatedness in some cases but no relatedness among *K. oxytoca* isolates.

**Conclusion:**

High prevalence of class 1 integron carrying gene cassettes confirms that integron-mediated antimicrobial gene cassettes are important in *Klebsiella spp*. resistance profile. Clone diffusions of MDR *Klebsiella spp*. which harbor class 1 integrons have threaten the potential in the resistance development in our clinical settings.

## INTRODUCTION

*Klebsiella spp*. are responsible for numerous diseases in different countries around the world (1, 2). The most prevalent species, *Klebsiella pneumoniae*, is one of the most prevalent agents of nosocomial infections which displays multidrug resistance (MDR) phenotype (2, 4). In the few past years transferable elements, plasmids and integrons, have been studied in microorganism particularly in Gram negative bacteria (5, 6). They transfer antibacterial gene cassettes which confer resistance to different antibiotics (7, 8).

To date, ten classes of integrons have been introduced, but in clinical isolates class 1 is the most prevalent. They have a potential to transfer various gene cassettes as antibiotic resistance agents (9). Over three hundred different cassette arrays were identified which are able to be flanked by the 5’-CS and 3’-CS regions (10). They confer resistance to all beta-lactam antibiotics, aminoglycosides, trimethoprim, rifampin and antiseptics.

In this study, we investigate the prevalence of class 1 integrons and integrated gene cassettes in *Klebsiella spp*. isolated from hospitalized and outpatients from Besat Hospital of Kurdistan, in the west part of Iran. Resistant to at least three classes of antimicrobial agents is considered as MDR isolates (10). Besides, we consider the rate of resistance in *Klebsiella spp*. and their relationships in terms of clonality relatedness.

## MATERIALS AND METHODS

Hospital setting and study population. Besat university hospital is a 300 bed referral hospital in the Sanandaj city in Kurdistan province, west of Iran. During 2009, approximately, 14400 individuals were admitted to this hospital. Moreover, outpatients refer to the clinical diagnosis laboratory of Besat hospital for routine tests.

### Bacterial isolates and data collection

During January - December 2009, the referred specimens were cultured on 5% sheep Blood Agar and EMB (Merck, Germany). Sterile body fluids (e. g. cerebrospinal fluid) were initially cultured in enrichment media, Thioglycollate broth (Merck, Germany) and Chocolate agar. They were then subcultured into the prior mentioned media. All Gram negative isolates were identified by API20E (BioMérieux, Marcy l'Etoile, France). Because of few suspicious reactions of API20E (e. g. Urease), biochemical activities were proved by individual macrotube differential biochemical tests (12). Only one isolate from each patient was included in this study. Age, sex, previous hospitalization, ward of patient and type of specimens (e. g. blood, urine, tracheal aspirate and so on) were recorded. Also, samples related to the instruments associated with the patients were collected. Isolated gram negative bacteria from such samples were characterized as well as patient's isolates. Isolated *Klebsiella spp*. were stored in Muller Hinton Broth (Merck, Germany) containing 18% of glycerol (Merck, Germany) at -80°C until used.

### Disk diffusion testing

The ten following antibiotic disks (MAST, UK) were tested for all isolates: aztreonam (30 µg), ceftriaxone (30 µg), cefepime (30 µg), ceftazidime (30 µg), cefotaxime (30 µg), imipenem (10 µg), meropenem (10 µg), amikacin (30 µg), gentamicin (10 µg) and ciprofloxacin (5 µg). The breakpoints for antibiotic susceptibility were determined according to the guidelines of the Clinical and Laboratory Standards Institute (CLSI) (13). *Escherichia coli* ATCC 25922 was used as negative control strain.

### Minimum inhibitory concentration test (MIC)

Three antibiotic powders from three different classes, ceftazidime, gentamicin and ciprofloxacin (Exir, Iran) were used to display whether isolates are MDR or not. Resistant to at least three classes of antibacterial agents considered as MDR isolates (11).

The MICs of all antibiotics against *Klebsiella spp*. were determined using microdilution method (14). Briefly, three above antibacterial powders was solved in the proper solvent, saturated NaHCO3, distilled water and 0.1N HCL, respectively. These solvents do not affect bactericidal effect of antibiotic powders. Then, a serial dilution prepared and dispersed in 96 well plates and inoculated with 5×10^5^ cfu.ml^−1^bacteria per well. After 18-20 hours in 35°C incubation results were recorded. Guidelines by CLSI (13) were considered as the reference. *E. coli* ATCC 25922 and *Pseudomonas aeruginosa* 27853 were used as control strains.

### DNA extraction and PCR for detection of intI1 and IVR

DNA of freshly 20 hours bacterial colonies were extracted by DNeasy Blood & Tissue Kit (QIAGEN, Germany) according to the manufacturer's instructions to extract bacterial DNA. DNA concentrations were determined spectrophotometrically of OD260/280 rate. PCR amplification was performed by using commercial kits of PCR (Bioneer, AccuPower PCR PreMix96 tubes, Korea). For amplification of *intI1* following primers were used, 5’-ATCATCGTCGTAGAGACGTCGG-3’ as forward and 5’-GTCAAGGTTCTGGACCAGTTGC-3’ as reverse (15). The gene cassettes inserted in the variable regions of class 1 integrons (IVR) were amplified using the primer pairs 5’-CS (5’-GGCATCCAAGCAGCAAG-3’) and 3’-CS (5’- AAGCAGACTTGACCTGA-3’) (16). Amplification was done by a thermocycler (Eppendorf Mastercyclers, MA) using the following program: initial denaturation at 94°C for 5 min and 35 cycles of 30 s at 94°C, 30 s at 55°C, and 2 min at 72°C, with a final extension for 5 min at 72°C (17). PCR products were purified using the QIAquick PCR Purification kit (QIAGEN Inc., Valencia, CA) and were subjected to direct sequencing by the ABI Capillary System (Macrogen Research, Seoul, Korea). The resistance gene cassettes in class 1 integrons were analyzed using the online BLAST of NCBI website software (http://www.ncbi.nlm.nih.gov/BLAST/).

### Restriction Fragments Length Polymorphism (RFLP)

To detect the contents of amplified IVRs, RFLP were exerted. *Alu* I (Fermentas, Vilnius, Lithuania) was used as digesting restriction endonuclease. Briefly, each 20 µl of the restriction mixture contained 2 µl (20 U) of enzyme, 8 µl of PCR-amplified product, 1 µl of enzyme buffer and 9 µl of double-distilled water. As manufacturer's guidelines, restriction mixtures were incubated at 37°C for one hour and electrophoresed on 2% agarose gel at 40V. Different RFLP patterns were subjected for sequencing.

### Nucleotide sequence accession number

Qiaquick Columns (Qiagen, Crawley, UK) were used to purify the PCR products obtained for IVRs from gel. The purified amplicons were sequenced using the ABI Capillary System (Macrogen Research, Seoul, Korea). Sequences were blasted by online BLAST software (http://www.ncbi.nlm.nih.gov/BLAST/). After these analyses, sequences were submitted to the EMBL/GenBank database (www.ncbi.nlm.nih.gov).

### Genotyping by pulsed-field gel electrophoresis (PFGE)

The PFGE method was performed with the standardized CDC protocol for MDR isolates (18). Electrophoresis was carried out in 0.5 × TBE buffer in pulsed field electrophoresis CHEF-DR III system (Bio Rad, Hercules, California, USA) with use of the restriction endonuclease *Xba*I (Fermentas, Vilnius, Lithuania). DNA was subjected to electrophoresis in a 1% agarose gel at 6 V/cm for 21 h at 14°C, with the pulse time ramped linearly from 2.2s to 54.2s. The molecular size marker included for comparison was *Salmonella choleraesuis* serotype *Branderup H9812* that was digested by *Xba* I, as well. Following electrophoresis, the gels were stained with ethidium bromide and visualized under ultraviolet light. The banding patterns were analyzed by the means of Gelcompare II software (Applied Math, Sint-Maten-Latem, Belgium) and interpreted based on the criteria described by Tenover et al. (19).

## RESULTS

### Bacterial species

During 2009, about 17890 clinical specimens referred to the Microbiology ward of the laboratory of Besat hospital. Among the large amount of specimens, the most frequent specimen were urine cultures (8172, 45.7%), followed by blood cultures (5190, 29%). 1110 episodes were characterized as Gram negative (778, 70%) and Gram positive (332, 30%). Among all Gram negative isolates 35 were identified as *Klebsiella spp*. (3.1%), of which *Klebsiella pneumonia* was the most (29, 83%) followed by *Klebsiella oxytoca* (6, 17%) (Table 1). No other *Klebsiella* species were isolated. 25 of *K. pneumonia* were isolated from hospitalized patients and 4 were from outpatients. Furthermore, one *K. oxytoca* was isolated from hospitalized and 5 were from outpatients.

**Table 1:** *Klebsiella* isolates and source of isolation.

Source	*K. pneumoniae* (n = 29)		*K. oxytoca* (n = 6)	

	Non-MDR	MDR	Non-MDR	MDR
**Urine**	4	9	3	3
**Blood**	1	1	0	0
**Trachea**	0	9	0	0
**Wound**	2	1	0	0
**Others**	1	0	0	0
**Total**	9	20	3	3

Abbreviation: MDR, multidrug resistant; non-MDR, non multidrug resistant.

### Antibiotic susceptibility testing

All of the *Klebsiella spp*. isolates were susceptible to imipenem (100%) and meropenem (100%) (Table 2). Conversely, ceftriaxone was the least efficient antibacterial agent (17%). Besides, high rate of resistance was seen for the other beta-lactam groups, including aztreonam (20%), ceftazidime (20%) and cefotaxime (20%). Gentamicin (31%) and amikacin (37%), were partly efficient. According to the Antibiogram profile, 23 out of 35 *Klebsiella spp*. isolates were MDR. Of which, 20 MDRs were *K. pneumoniae* and three were *K. oxytoca* (Table 1).

**Table 2:** Antibiotic profile of all *Klebsiella spp*. isolated in this study.

	Susceptible no. (%)	Intermediate no. (%)	Resistant no. (%)
**IMI**	35(100)	0 (0)	0 (0)
**MEM**	35(100)	0 (0)	0 (0)
**CAZ**	7(20)	0 (0)	28(80)
**CTX**	7(20)	0 (0)	28(80)
**CRO**	6(17)	0 (0)	29(83)
**CPM**	7(20)	0 (0)	28(80)
**CIP**	11(31)	3(8)	21(61)
**GM**	11(31)	4(11)	20(58)
**AK**	13(37)	4(11)	18(52)
**ATM**	7(20)	0 (0)	28(80)

Abbreviations: IMI, imipenem; MEM, meropenem; CAZ, ceftazidime; CTX, cefotaxime; CRO, ceftriaxone; CPM, cefepime; CIP, ciprofloxacin; GM, gentamicin; AK, amikacin; ATM, aztreonam.

All 23 MDR *Klebsiella spp*. isolates were subjected for MIC of three different antibacterial classes (Table 3). Results revealed high rate of resistance to ceftazidime and gentamicin.

**Table 3:** Characteristics of integrons and their integrated gene cassettes identified in the integron-carrying MDR *Klebsiella spp*. isolates.

MDR Isolates name	Isolation source	Ward of isolation	MIC(µg mL^−1^)			Length of IVRs (bp)	Integron gene cassettes

			CIP	CAZ	GM		
HSZKP1	W/C	ICU2	2	64	32	400, 800	arr-5 / aacA4-orfD
HSZKP2	U/ C	IM	64	128	64	400, 800, 2300	arr-5 / aacA4-orfD / dfrA17-aadA5
HSZKP3	Tr	ICU1	4	64	32	400, 800, 2300	arr-5 / aacA4-orfD / dfrA17-aadA5
HSZKO6	U/C	E	<0.25	1	1	400, 800	arr-5 / aacA4-orfD
HSZKO7	U/C	OP	<0.25	<0.5	<0.5	800	aacA4-orfD
HSZKP8	U/C	ICU2	<0.25	16	2	800, 2300	arr-5 / aacA4-orfD / dfrA17-aadA5
HSZKP9	Tr	ICU2	4	32	8	400, 800, 2300	arr-5 / aacA4-orfD / dfrA17-aadA5
HSZKP19	U/C	ICU2	0.25	32	8	400, 800, 2300	arr-5 / aacA4-orfD / dfrA17-aadA5
HSZKP32	Tr	ICU2	4	128	64	400, 800, 2300	arr-5 / aacA4-orfD / dfrA17-aadA5
HSZKP33	Tr	ICU1	2	128	64	400, 800, 2300	arr-5 / aacA4-orfD / dfrA17-aadA5
HSZKP43	Tr	ICU2	4	16	32	400, 800, 2300	arr-5 / aacA4-orfD / dfrA17-aadA5
HSZKP44	Tr	ICU1	2	256	128	400, 800	arr-5 / aacA4-orfD
HSZKP45	U/ C	ICU1	2	32	8	400, 800, 2300	arr-5 / aacA4-orfD / dfrA17-aadA5
HSZKP54	Tr	ICU2	<0.25	128	32	400, 800, 2300	arr-5 / aacA4-orfD / dfrA17-aadA5
HSZKP71	U/ C	E	8	128	16	400, 800, 2300	arr-5 / aacA4-orfD / dfrA17-aadA5
HSZKP89	U/C	OP	32	16	4	400, 800, 2300	arr-5 / aacA4-orfD / dfrA17-aadA5
HSZKP90	Tr	NS	32	128	64	400, 800, 2300	arr-5 / aacA4-orfD / dfrA17-aadA5
HSZKP91	Tr	ICU1	32	256	64	400, 800, 2300	arr-5 / aacA4-orfD / dfrA17-aadA5
HSZKP93	W/C	PICU	<0.25	16	2	400, 800	arr-5 / aacA4-orfD
HSZKP95	U/C	OP	1	2	1	400, 800	arr-5 / aacA4-orfD
HSZKP96	BS/C	OP	0.5	16	8	400, 800, 2300	arr-5 / aacA4-orfD / dfrA17-aadA5
HSZKP97	B/C	PICU	<0.25	2	2	400, 800	arr-5 / aacA4-orfD
HSZKO98	U/C	ICU1	<0.25	2	0.5	800	aacA4-orfD

Abbreviations resistance criteria: W/C, Wound culture; U/C, Urine culture; Tr, Trachea; BS/C, Brain shunt culture; B/C, Blood culture; IVRs, internal variable regions; ICU, Intensive Care Unit; PICU, Pediatrics Intensive Care Unit; OP, Outpatient; E, Elective; IM, Internal Medicine; NS, Neurosurgery. K. p, *Klebsiella pneumonia;* K. oxy, *Klebsiella oxytoca*; Break points for MIC: CIP= susceptible (≤1 µg mL^−1^), intermediate (2 µg mL^−1^) & resistant (≥4 µg mL^−1^); CAZ = susceptible (≤4 µg mL^−1^), intermediate (8 µg mL^−1^) & resistant (≥16 µg mL^−1^); GM= susceptible (≤4 µg mL^−1^), intermediate (8 µg mL^−1^) & resistant (≥16 µg mL^−1^).

### PCR for intI1, IVRs and RFLP results

In all of the MDR *Klebsiella spp*., *intI1* (100%) were detected. Integrated gene cassettes into the class 1 integrons were characterized in different sizes from 100 to 2300 bp (Table 3). The most prevalent IVRs were 400, 800 and 2300 bands. Most of isolates contain numerous IVR bands. All of the isolates (100%) had 800 bp bands, 20 out of 23 (87%) had 400 bp bands and 15 out of 23 (65%) isolates had 2300 bp bands. PCR products were digested by RFLP-PCR for polymorphism studies (data not shown). As expected, isolates had different patterns.

### Sequencing and accession numbers

Three prevalent bands 400, 800 and 2300 bp were sequenced and blasted in NCBI data bank. Sequencing results were as follows respectively: *Arr-5 (arr-5)* which codes ADP-ribosyl transferases; *aacA4- OrfD* codes Aminoglycoside (6’) N-Acetyltransferase & unknown function; *dfrA17- aadA5* codes dihydrofolate reductase and aminoglycosides adenylyltransferase. Accession numbers for 400, 800 and 2300 bp gene cassette in this study were JN222800.1, JN222799.1 and JN222798.1, respectively.

### PFGE analyzes

**Fig. 1:**
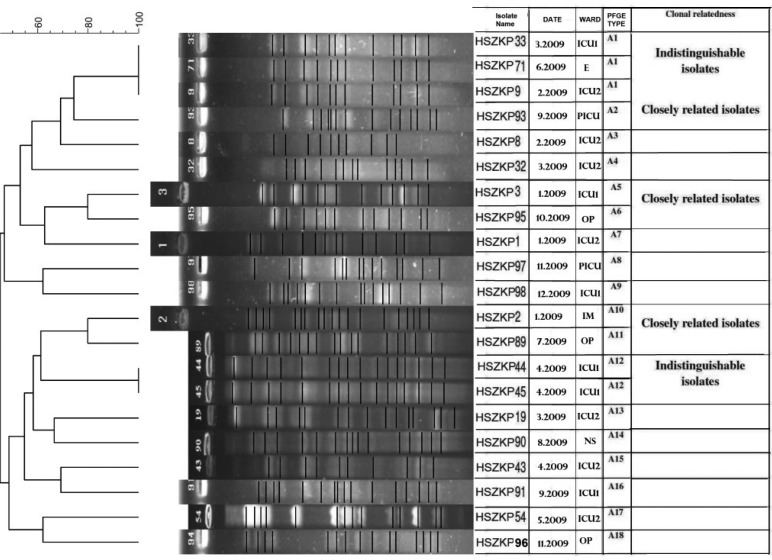
PFGE typing of MDR K. pneumoniae isolates with XbaI. 18 clusters of MDR K. pneumoniae isolates were distinguished by dendrogram (Gelcompare II software). Abbreviations: ICU, Intensive Care Unit; PICU, Pediatrics Intensive Care Unit; OP, Outpatient; E, Elective; IM, Internal Medicine; NS, Neurosurgery.

Separately, 20 *K. pneumoniae* and three *K. oxytoca* MDR isolates were analyzed by PFGE (Fig. 1). There was no relatedness among *K. oxytoca* isolates (not shown), but in *K. pneumonia* there were 18 clusters and clonality relatedness in some cases.

## DISCUSSION

In Besat teaching hospital, wide varieties of specimens provided a good source to view and interpret epidemiology of *Klebsiella* species. However, due to of lack of careful surveillance for detection of bacterial pathogens, we had probably missed some cases of *Klebsiella*. Our data suggests that *K. pneumoniae* is the most common isolate in the class of *Klebsiella* which is in agreement with other studies (9, 20). Also, six *K. oxytoca* were isolated. Of which, 5 out of 6 were from outpatients. Our results are the same as other authors’ (9, 20). Twent-five out of 29 of *K. pneumoniae* were isolated from the hospitalized patient. It supports the fact that this species has a potential to colonize patients by hospital stay (nosocomial infections) rather than outpatients (community acquired infections). In contrast, this is true about *K. oxytoca*. MDR *K. oxytoca* isolates were resistant to gentamicin, ceftriaxone and nalidixic acid (data not shown). They were susceptible to ceftazidime, ciprofloxacin and carbapenems.

Most of the MDR isolates belonged to patients in ICU which admits patients with risk factors (21). In our study, all isolates from tracheal specimens were MDR with high rates of MIC (Tables 1 & 3). Long hospital stay and antibiotic pressure select resistant strains which were colonized in susceptible patients (4). In these conditions physicians have limited drug choices. High percentage of resistant to ceftazidime, aztreonam and the other beta lactams show the high rate of beta lactam prescription. Also, aminoglycosides are used in combination therapy with beta- lactam antibiotics. Therefore, it is expected to reveal high rate of resistance to aminoglycosides as well as beta-lactams. Although, sequencing analyzes show integrated gene cassettes related to aminoglycoside resistant in most isolates, however, in total there are medium rates of resistance for aminoglycosides (gentamicin, 58% and amikacin 52%). Among antibacterials imipenem and meropenem, hospital drug choices, are the most functional. Fortunately, all isolated *Klebsiella spp*. were susceptible to imipenem and meropenem. Limited use of these two antibacterial drugs in this hospital is the reason of low rate of susceptibility. In 2008, Ramazanzadeh reported the same results in this hospital (22). Studies from other parts of Iran reported lower resistance patterns (23–26). However, Nematzadeh et. al. showed imipenem resistant *K. pneumoniae* isolates (2.1%) while resistance percentage for other antibacterial classes were lower than our isolates (27). Moreovere, Shahcheraghi et. al. have recently reported carbapenem resistant isolates that carry *bla*_NDM-1_ (28). Again, it should be stressed that our results is due to high prescribed cephalosporins and limited use of carbapenems.

Amplification for IVR of class 1 integron showed different bands that revealed integration of class 1 integron in different sites in the bacterial chromosome. Integron-typing, PCR-RFLP, represented different patterns which can be concluded that there were different gene cassettes in the same bands. In this study, we have been evaluated the most prevalent bands, therefore, more studies on all PCR bands are required to find out what kind of other gene cassettes are integrated. Other surveys confirmed diversity of inserted gene cassettes, in other parts of the world in different bacterial strains (20, 20). In Iran, different impressive studies characterized the *dfr*A25 (0.75 kb), *aad*A1 (1 kb), *aad*A2 (1 kb), *bla*(PSE1) (1.2 kb) *aad*A6-orfD (1.3 kb) gene cassette arrays which are different from our gene cassettes (29–31). It can be deduced that different regions are the territory of some gene cassettes. It should be worked on other regions to verify this hypothesis.

Distribution of integrated gene cassettes among all isolates during one year in hospitalized and outpatients are the same in many cases (Table 3). In other word, there is no difference of the prevalence of gene cassettes along with the time course. It can be deduced that spreading gene cassettes in the geographical region of this study are endemic. Besides, PFGE analysis shows the same deduction, as illustrated in picture 1.

According to the PFGE results, different clones have the same pattern of integrated gene cassettes (Fig. 1) which reflect dissociation of spreading of integrated gene cassettes by one or more clones. More investigations are required to find putative source of wide distribution of class 1 integrons. It is difficult to prove whether patients were colonized or infected by *Klebsiella spp*., especially, where there are not previous epidemiologically records.

In conclusion, the data presented herein illustrate the high rate of antibacterial resistance in *K. pneumoniae* and diverse integrated gene cassettes related to class 1 integrons. Different patterns of PCR-RFLP and PFGE among hospitalized and outpatients show wide distribution of integrons which reflect the issue in the case of endemic. Further investigations are required for analyzing other PCR bands on a wider range of bacterial collection to detect other integrated gene cassettes.
