# Errata

**Published:** 2013-06

Iranian Journal of Microbiology, 5 (No. 1) 2013. In the article titled:

Molecular characterization of class 1 integrons and gene cassettes in multidrug resistant (MDR) Klebsiella spp. isolated from hospitalized and outpatients in Iran

The authors’ names should be corrected as follows:

Himen Salimizand^1^, Fereshteh Shahcheraghi^2^*, Enayatollah Kalantar^1^, Farzad Badmasti^2^, Seyed Fazlollah Mousavi^1^

